# COVID-19 death risk predictors in Brazil using survival tree analysis: a retrospective cohort from 2020 to 2022

**DOI:** 10.1186/s12939-024-02101-x

**Published:** 2024-02-20

**Authors:** Carlos Martins Neto, Maria dos Remédios Freitas Carvalho Branco, Alcione Miranda dos Santos, Bruno Luciano Carneiro Alves de Oliveira

**Affiliations:** Postgraduate Program in Public Health, Department of Public Health, Universidade Federal do Maranhão, São Luís, Maranhão Brazil

**Keywords:** COVID-19, SARS-CoV-2, Risk factors, Epidemiology, Survival tree analysis, Interaction

## Abstract

**Purpose:**

This study analyses the survival of hospitalized patients with Severe Acute Respiratory Syndrome (SARS) due to COVID-19 and identifies the risk groups for death due to COVID-19 from the identification of potential interactions between its predictors.

**Methods:**

This was a retrospective longitudinal study with data from 1,756,917 patients reported in the Influenza Epidemiological Surveillance Information System from 26 February 2020 to 31 December 2022. In this study, all adult and older (≥ 20 years) patients were hospitalized with SARS due to COVID-19, with death as the outcome. Survival tree analysis was used to identify potential interactions between the predictors. A model was built for each year of study.

**Results:**

Hospital lethalitywas 33.2%. The worst survival curve was observed among those who underwent invasive mechanical ventilation and were aged 80 years or older in the three years of the pandemic. Black and brown race/color were predictors of deaths in the years 2020 and 2021 when there was greater demand from the health system due to the greater number of cases.

**Conclusion:**

By applying survival tree analysis we identified several numbers of homogeneous subgroups with different risks for mortality from COVID-19. These findings show the effects of wide inequalities of access by the population, requiring effective policies for the reduction and adequate management of the disease.

## Introduction

Brazil is among the countries most affected by the COVID-19 epidemic, with a high number of cases, hospitalizations, and deaths. Many cases have evolved into the most severe forms of the disease, dependent on hospitalization and intensive care, especially among the most vulnerable patients and those with risk factors associated with the severity of COVID-19. This condition makes Brazil one of the world’s highest proportions of hospital deaths and the lowest hospital survival rates [1].

Data from a systematic review indicate that among cases of COVID-19, the rate of admission to the Intensive Care Unit (ICU) can be 21%, and 69% require mechanical ventilation [2]. Mortality for patients who were admitted to the ICU can be 28.3% and 43% for mechanically ventilated patients [2]. Hospital mortality from COVID-19 in Brazil (38%) [3] was higher than in other countries, such as Germany (22%) [4] and the United States (35.4%) [5] in the first year of the pandemic.

Extensive literature describes several individual risk factors and those related to the network of health services that can lead to the worsening of COVID-19 and generate a higher risk of death, even in hospital care. Among the individual risk factors are black or brown color/race, advanced age, male sex, and presence of comorbidities (especially obesity, diabetes, hypertension, cancer, and chronic kidney disease) [5–8].

A survey in Brazil showed that an increase in the number of serious cases in cities in the interior of Brazil caused an overload of small hospitals with few qualified human resources and ICU beds to manage this increase in demand, and the consequent need to transport these patients to large capitals overloads the health system throughout the country [9].

This overload also occurred due to the denialist stance of the Federal Government at the time, which resulted in a non-uniform and integrated response to COVID-19 throughout Brazil, even using treatments without robust scientific evidence of their effectiveness [10]. In contrast to the Federal Government, many municipal and state governments, media sectors, political parties, and the judiciary based their actions on scientifically based measures, in line with the efforts of scientific organizations that mobilize for the strengthening of the Unified Health System (SUS) [10]. Unfortunately, this did not completely prevent the disastrous consequences we faced, with more than 700,000 people dead from the disease [11].

However, despite the large volume of knowledge generated since its discovery in December 2019, there is still diversity between countries, regions, and cities in coping responses to COVID-19, with serious repercussions on the survival of these patients. Much of the research already carried out has not yet revealed which characteristics of patients can interact with each other and explain the survival situation of patients with COVID-19 in Brazil since previous studies focused on risk factors in isolation.

Therefore, this study sought to analyze the survival of hospitalized patients with Severe Acute Respiratory Syndrome (SARS) due to COVID-19 and to identify the groups at risk of death due to COVID-19 based on the identification of potential interactions between their predictors.

## Methods

This was a retrospective longitudinal study with data from 1,756,917 patients notified in the Influenza Epidemiological Surveillance Information System (SIVEP-Gripe) from 26 February 2020 to 31 December 2022. The SIVEP-Gripe is an information system created by the Ministry of Health to record cases and deaths from SARS and COVID-19 in Brazil. In it, the notification of COVID-19 is compulsory and receives information from patients in public and private hospitals, as well as about those who died without hospitalization. The data were extracted from the *OpenDataSUS* (https://opendatasus.saude.gov.br/) website on 1 May 2023.

In this study, all patients aged 20 years or older (adults and elderly) who had a final classification of SARS due to COVID-19 and at least one day of hospitalization were included. Those with missing information or typing errors on the date of hospitalization, discharge date, or information on the evolution of the case (death or discharge) were excluded.

### Variables

In this study, the following variables were evaluated: sociodemographic - sex (men, women), age (20 to 39, 40 to 59, 60 to 79, and 80 years or older), race/color (White, Black, Yellow, Brown, Indigenous), country’s macro-region (Midwest, North, Northeast, South, Southeast), clinical data, Intensive Care Unit Admission (Yes, No), Mechanical Ventilation (Invasive, Non-Invasive, No), and Risk Factor (No, One factor, Two factors, Three or more).

The risk factor variable refers to the number of comorbidities reported by the patient at the time of hospitalization, which includes the following risk factors: postpartum women, chronic cardiovascular disease, chronic hematological disease, chronic liver disease, asthma, diabetes mellitus, chronic neurological disease, chronic lung disease, immunosuppression, chronic kidney disease, and obesity.

For the survival analysis, the outcome (failure) was the occurrence of hospital death within a maximum of 90 days of hospitalization and the survival time, defined from the date of admission to the date of evolution (discharge or death). All individuals who were discharged within 90 days or remained hospitalized after this period were excluded.

### Data analysis

All analyses were performed using R software (http://www.r-project.org/). To deal with the missing values of some variables, we used a single imputation with the Fully Conditional Specification (FCS) method implemented with the MICE Package [12].

After imputation, descriptive analyses (proportion, mean, and standard deviation) of the variables considered in the study were performed.

The hospital fatality rate was calculated as follows:

$$\frac{{Number\,of\,hospital\,deaths\,due\,to\,COVID - 19\,SRAG\,in\,the\,period}}{{Total\,number\,of\,reported\,hospitalizations\,due\,to\,COVID - 19\,SRAG\,in\,the\,period}}\, \times 100$$

The survival curve of the included cases was constructed using the Kaplan-Meier method. To identify different groups at risk of death from COVID-19, based on the interactions between the socioeconomic, demographic, and clinical characteristics of hospitalized patients, the survival tree (ST) method was used. The survival tree is a nonparametric technique that incorporates tree-structured regression models. From the survival tree, individuals were grouped according to their survival time and based on the independent variables. Thus, this technique allows the automatic detection of complex interactions between variables without the need to specify them a priori [13].

For the construction of the survival tree, survival time was defined as the time (days) of hospitalization. The patient’s sociodemographic and clinical characteristics were included in the tree as independent variables, and the patient’s status as a dependent variable: 1 (one) if death occurred within 90 days and 0 (zero) if there was no death within that period (discharge or hospital stay longer than 90 days).

ST groups patients according to survival time and independent variables, and the sample is divided into subgroups (nodes) based on an independent variable. First, the initial node (root node) of the tree is obtained, child nodes are created, and this procedure is repeated until the terminal node is reached [13]. This method automatically detects complex interactions between variables without the need to specify them in advance. ST was implemented in the statistical program R, using the *Survival, LTRCtrees* and *Party.kit* packages.

The risk of death was determined at each terminal node of the tree, and Kaplan-Meier curves were constructed for each terminal node. The minimum criterion for node division was defined as *P* < 0.05.

After defining the tree and the number of terminal nodes contained in the tree, a categorical variable was created to specify which terminal node the patient was included. Thus, considering that the tree contained *k* terminal nodes, the categorical variable comprised *k* categories (groups at risk of death). For obtaining the Hazard Ratio (HR) of death events at each terminal node, a univariate Cox model was fitted, having as an independent variable only the categorical variable that specifies the node to which the patient belongs since this variable was obtained from the independent variables considered in the tree. The terminal node-containing patients with the lowest risk of death were considered as the reference category. A model was built for each year of study.

Appraisal by the Ethics Committee of research involving human beings was not necessary, as this research was prepared only with secondary public data available online at official electronic sites. These databases do not contain personal or household identification data, which guarantees respect for the secrecy and privacy of the research participants’ information [14].

## Results

Among the 1,756,917 hospitalized patients with COVID-19 assessed in this study, 585,914 (33.2%) died.

A statistically significant difference was observed between all covariates under study and deaths due to COVID-19 (*p* < 0.001). In 2020 lethality was slightly higher in men (34.7%), aged 80 years or older (60.6%), of black race/color (37.3%), from the northeast region (42.3%) (Table 1). In 2021 lethality was slightly higher in women (33.7%), aged 80 years or older (59.1%), of black race/color (37.2%), and from the north region (40.7%). Similar results were found in 2022, with the difference being that the Northeast region had the highest lethality rate (35.6%) (Table 1).

**Table 1:** Sociodemographic characteristics of hospitalized adults and older adults with SARS due to COVID-19 in Brazil in 2020–2022

Variables	2020		2021		2022	
	Censored (*n* = 363,710) f (%)	Deaths (*n* = 186,112) f (%)	Censored (*n* = 685,881) f (%)	Deaths (*n* = 344,404) f (%)	Censored (*n* = 123,585) f (%)	Deaths (*n* = 53,225) f (%)
**Sex**						
Woman	162,209 (67.2)	79,141 (32.8)	301,204 (66.3)	152,799 (33.7)	59,692 (67.7)	28,523 (32.3)
Man	201,501 (65.3)	106,971 (34.7)	384,677 (66.8)	191,605 (33.2)	63,893 (72.1)	24,702 (27.9)
**Age (years)**						
20 to 39	61,096 (89.3)	7,332 (10.7)	138,832 (85.3)	23,844 (14.7)	15,168 (89.9)	1,712 (10.1)
40 to 59	142,732 (80.0)	35,725 (20.0)	307,073 (74.9)	102,697 (25.1)	24,402 (79.1)	6,456 (20.9)
60 to 79	127,301 (57.8)	92,887 (42.2)	197,656 (55.8)	156,750 (44.2)	48,412 (68.7)	22,067 (31.3)
80 or more	32,581 (39.4)	50,168 (60.6)	42,320 (40.9)	61,113 (59.1)	35,603 (60.8)	22,990 (39.2)
**Race/Color**						
White	179,547 (67.9)	84,891 (32.1)	370,237 (67.2)	180,790 (32.8)	73,223 (70.5)	30,616 (29.5)
Black	23,266 (62.7)	13,815 (37.3)	33,791 (62.8)	20,038 (37.2)	5,751 (66.8)	2,854 (33.2)
Yellow	7,036 (68.3)	3,271 (31.7)	11,241 (70.7)	4,666 (29.3)	2,010 (71.6)	799 (28.4)
Brown	150,324 (64.7)	82,162 (35.3)	265,562 (66.1)	136,311 (33.9)	41,544 (69.1)	18,542 (30.9)
Indigenous	3,537 (64.2)	1,973 (35.8)	5,050 (66.0)	2,599 (34.0)	1,057 (71.9)	414 (28.1)
**Country’s macro-region**						
Southeast	191,516 (67.6)	91,830 (32.4)	348,052 (67.0)	171,122 (33.0)	65,561 (69.7)	28,464 (30.3)
South	57,363 (70.9)	23,562 (29.1)	139,912 (68.6)	64,050 (31.4)	26,421 (72.3)	10,107 (27.7)
Midwest	38,559 (70.1)	16,447 (29.9)	75,097 (69.3)	33,235 (30.7)	12,105 (74.1)	4,225 (25.9)
North	23,460 (60.2)	15,489 (39.8)	34,269 (59.3)	23,480 (40.7)	4,530 (67.7)	2,157 (32.3)
Northeast	52,812 (57.7)	38,784 (42.3)	88,551 (62.8)	52,517 (37.2)	14,968 (64.4)	8,272 (35.6)

In the three years studied, we observed a higher lethality among patients with three or more risk factors, those who were admitted to the ICU, and required invasive mechanical ventilation. The average length of stay was shorter in 2020 (12.7 ± 13.7 days) among those who died (Table 2).

**Table 2:** Clinical characteristics of adults and older adults hospitalized with SARS due to COVID-19 in Brazil in 2020–2022

Variables	2020		2021		2022	
	Censored f (%)	Deaths f (%)	Censored f (%)	Deaths f (%)	Censored f (%)	Deaths f (%)
**Risk Factor**						
No	177,748 (75.2)	58,584 (24.8)	389,685 (74.8)	131,339 (25.2)	51,430 (75.7)	16,504 (24.3)
One factor	112,367 (63.6)	64,348 (36.4)	188,222 (62.3)	114,033 (37.7)	40,019 (68.7)	18,264 (31.3)
Two factors	57,727 (56.2)	45,064 (43.8)	85,427 (54.2)	72,285 (45.8)	23,311 (64.9)	12,588 (35.1)
Three or more factors	15,868 (46.7)	18,116 (53.3)	22,547 (45.7)	26,747 (54.3)	8,825 (60.1)	5,869 (39.9)
**UCI Admission**						
Yes	93,976 (43.9)	120,318 (56.1)	166,633 (42.1)	229,252 (57.9)	31,800 (50.0)	31,850 (50.0)
No	269,734 (80.4)	65,794 (19.6)	519,248 (81.8)	115,152 (18.2)	91,785 (81.1)	21,375 (18.9)
**Mechanical Ventilation**						
No	117,984 (86.0)	19,139 (14.0)	138,700 (87.7)	19,517 (12.3)	48,613 (90.2)	5,252 (9.8)
Non-Invasive	220,966 (74.0)	77,623 (26.0)	496,339 (77.0)	148,538 (23.0)	68,524 (72.6)	25,895 (27.4)
Invasive	24,760 (21.7)	89,350 (78.3)	50,842 (22.4)	176,349 (77.6)	6,448 (22.6)	22,078 (77.4)
**Hospitalization time (days)**						
Mean (SD)	11.6 (13.4)	14.1 (13.9)	10.6 (11.8)	13.6 (12.3)	10.2 (12.4)	12.7 (13.7)
Median (Q1-Q3)	7.0 (4.0–13.0)	10.0 (5.0–18.0)	7.0 (4.0–12.0)	10.0 (5.0–18.0)	6.0 (3.0–12.0)	8.0 (4.0–16.0)
Minimum-Maximum	1.0–90.0	1.0–90.0	1.0–90.0	1.0–90.0	1.0–90.0	1.0–90.0

**Fig. 1:**
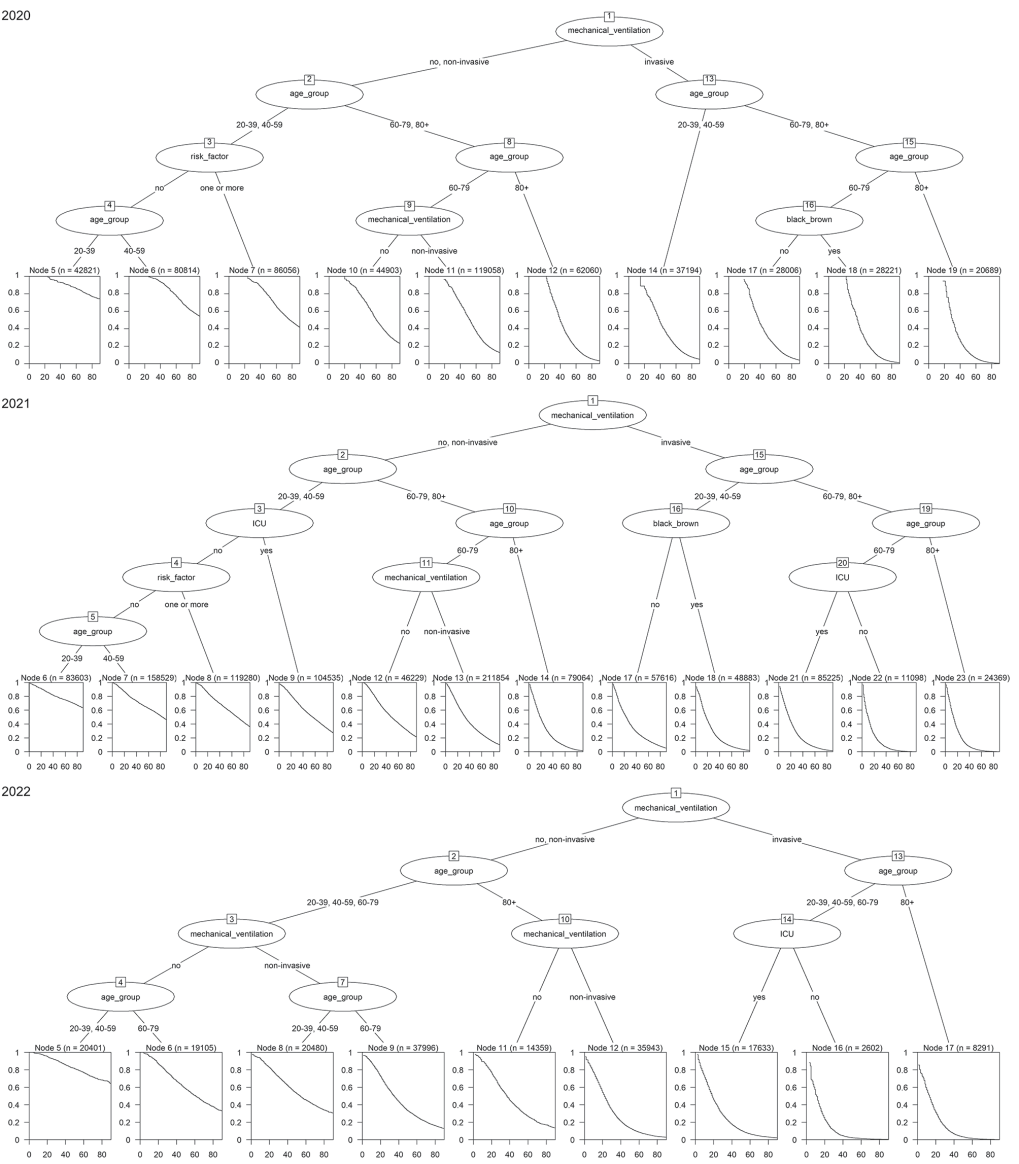
Survival tree for death events in adults and older people hospitalized for COVID-19 in Brazil, 2020–2022. **Notes**: Squares represent terminal nodes; the numbers (n) in the squares indicate the sample size; and the curves inside the squares show the estimated Kaplan-Meier survival of the subgroups. Circles represent the most significant variables for dividing the population into smaller groups.

The survival tree results are shown in Fig. 1. Mechanical ventilation, age, ICU stay, number of risk factors, and race were selected by tree to group the cases. The best cut-off points for these predictors were determined using the survival tree algorithm. Mechanical ventilation and age were considered the most important predictors of death from COVID-19.

The Kaplan-Meier curves for these groups are shown in Fig. 1. Ten groups were identified using the survival-tree algorithm in 2020. The curves of nodes 12, 14, 17, 18, and 19 were those with the worst survival curves showing the higher risk groups for death due to COVID-19 this year. The curves of the other nodes (5, 6, 7, and 10) had a higher probability of survival, that is, a lower risk of death from COVID-19. Node 19 had the highest risk of death from COVID-19 (HR: 16.20; 95%CI: 15.38–17.07). This group was composed of patients who used invasive mechanical ventilation, and 80 years or older. In 2021 twelve groups were identified using the survival-tree algorithm. The curves of nodes 14, 17, 18, 21, 22, and 23 were those with the worst survival curves and the curves of the other nodes (6, 7, 8, 9, and 12) had a higher probability of survival. The worst survival curve utilized the same variables as the previous year (HR: 12.58; 95%CI: 12.12–13.05). In 2022, only the use of invasive mechanical ventilation and age were identified as risk predictors for death by the survival tree, and only nine groups were identified using the survival-tree algorithm. Nodes 12, 15, 16, and 17 exhibited the poorest survival curves, while nodes 5, 6, 8, 9, and 11 had a higher probability of survival. However, the poorest survival curve was observed among those who underwent invasive mechanical ventilation, were younger than 80 years old, and did not require ICU admission (HR: 13.78; 95%CI: 12.81–14.82) (Table 3).

**Table 3:** Cox analysis of nodes identified using survival tree

2020		2021		2022	
Node	HR (95% CI)	Node	HR (95% CI)	Node	HR (95% CI)
5	Ref^1^	6	Ref^2^	5	Ref^3^
6	2.03 (1.92–2.14)	7	1.78 (1.71–1.85)	6	2.26 (2.09–2.46)
7	2.93 (2.78–3.09)	8	2.39 (2.30–2.48)	8	4.01 (3.70–4.34)
10	4.24 (4.02–4.48)	9	2.97 (2.87–3.09)	9	2.74 (2.53–2.96)
11	5.77 (5.48–6.07)	12	3.18 (3.05–3.31)	11	4.72 (4.39–5.07)
12	9.98 (9.47–10.50)	13	4.65 (4.48–4.81)	12	7.87 (7.33–8.45)
14	8.39 (7.97–8.84)	14	8.08 (7.80–8.38)	15	9.28 (8.65–9.97)
17	10.33 (9.81–10.88)	17	5.80 (5.60–6.02)	16	14.01 (12.88–15.24)
18	13.18 (12.51–13.88)	18	7.51 (7.25–7.79)	17	13.78 (12.81–14.82)
19	16.20 (15.38–17.07)	21	8.58 (8.28–8.89)		
		22	13.50 (12.96–14.06)		
		23	12.58 (12.12–13.05)		

**Notes**: ^1^ - No Mechanical or Non-Invasive Ventilation, No Risk Factor, 20 to 39 years; ^2^ - No Mechanical or Non-Invasive Ventilation, No ICU, No Risk Factor, 20 to 39 years; ^3^ - No Mechanical Ventilation, 20 to 59 years; Each node was compared with the column reference node

## Discussion

The results of this study showed that 33.2% of adult and older patients hospitalized in Brazilian hospitals in 2021 died and that there was a set of characteristics associated with the observed mortality. Survival time decreased with the length of hospital stay. Invasive mechanical ventilation, older age, ICU stay, and black or brown race/color were significant predictors of death from COVID-19.

Patients aged 60–79 years who used invasive mechanical ventilation and did not go to the ICU were the group with the highest risk among those presented in the survival tree. This indicates that the severity of lung impairment, in this case, is detected by the need for invasive support, which reduces the survival of these patients. Advanced age is a well-documented risk factor in the literature, and the older the age group, the higher the risk of patients with COVID-19 [5, 15].

It was also observed that older people over 80 years of age who did receive invasive ventilatory support had a higher risk than their younger peers. This result points to a possible non-institutionalization of the advanced support routine for older people with this need, increasing the mortality in this group. It is known that with the exponential increase in cases of COVID-19, there was saturation of the health system across the country, which meant that there was a shortage of ICU beds and, consequently, advanced ventilatory support in several regions [9]. This may have meant that older patients did not receive all the resources necessary for the proper management of their health conditions, which further exposes the vulnerabilities faced by the older population in the country.

Race/color is another determinant of mortality and survival in the Brazilian population, identified in the first two years of the pandemic. Thus, in situations of scarcer health resources, black and brown people become more vulnerable. A study with data from the 2013 National Health Survey that analyzed the factors associated with poor access to health services found that individuals with brown/black skin color, residing in the North or Northeast regions had a higher proportion of poor access to services [16]. Data on morbidity and mortality from COVID-19 according to race/color in Brazil and the United States indicate a greater impact of the disease on the black population. Even with the low completeness of data on race/color, which makes more robust research unfeasible, it is possible to verify that despite the greater hospitalization in the white population, the highest mortality occurred in the black and indigenous populations [17].

These data were confirmed in a previous study on mortality from COVID-19 in Brazil according to ethnic and regional variations, which observed an increase in mortality among brown and black people and those who live in the northern region [18]. Race/color is a determining factor in access to health services, especially in the ICU, which is an extremely necessary environment for the care of patients with more serious illnesses.

A literature review points out that racial inequalities in access to health services are long term and persist today. In brown and black populations, socioeconomic inequalities and inequalities in access to social and health services overlap, and the accumulation of these disadvantages affects living and health conditions [19].

Despite important advances in the Unified Health System (SUS), which defines better health outcomes in Brazil [20], there are still weaknesses in the quality of services offered. The complex public-private relationship in the provision of health services, associated with deep regional inequalities and the underfunding of the system, are still challenges that need to be overcome to improve the quality of the population’s health [21].

The presence of one or more risk factors proved to be variables that had less impact on the mortality of these patients than others, such as age. Thus, age seems to be the most significant factor contributing to the mortality of patients with COVID-19. In addition, the difficulty of properly diagnosing severity, which is determined by clinical and radiological manifestations, and the lack of beds in several regions of the country, as mentioned earlier, made this group much more vulnerable during the pandemic.

Immunization in Brazil began in January 2021, with older adults being priority groups during the vaccination campaign. The high risk of mortality in this group, even after vaccination, may be due to the drop in neutralizing antibodies among those immunized with *CoronaVac*, which was the vaccine most used in older adults at the beginning of the campaign [22] and/or the emergence of variants such as Delta, which have a higher lethality rate when compared to the original virus [23].

Although previous studies have shown the risk factors for death from COVID-19, the authors did not find any study that showed the effect of the interaction between risk factors.

This study had some limitations which must be mentioned. First, there may be biases in filling out patient information, which is common in all observational studies. Second, all the patients surveyed were hospitalized for SARS; therefore, it is necessary to consider that the mortality presented in this study is only in severe cases, thereby preventing the generalization of these results. Another point to be mentioned is related to missing data, which is also inherent to records in attendance forms and in large databases. However, an attempt was made to reduce this limitation by imputing data.

## Conclusions

Analysis of the results indicated that the use of invasive mechanical ventilation, ICU stay, advanced age, and black and brown race/color were important risk factors for death due to COVID-19. These findings highlight the effects of broad social and racial inequalities that make a population group more vulnerable to infection by the virus. They also highlight the requirement of effective policies aimed at reducing the poor access of the population to tests necessary for the correct diagnosis and management of the disease.

## Data Availability

All data used in this research is publicly available on the *OpenDataSUS* (https://opendatasus.saude.gov.br/) website.

## Abbreviations

ICU: Intensive Care Unit
SARS: Severe Acute Respiratory Syndrome
SIVEP: Gripe-Influenza Epidemiological Surveillance Information System
FCS: Fully Conditional Specification
ST: Survival Tree
SUS: Unified Health System
